# Villus Growth, Increased Intestinal Epithelial Sodium Selectivity, and Hyperaldosteronism Are Mechanisms of Adaptation in a Murine Model of Short Bowel Syndrome

**DOI:** 10.1007/s10620-018-5420-x

**Published:** 2018-12-20

**Authors:** Peggy Berlin, Johannes Reiner, Jakob Wobar, Karen Bannert, Änne Glass, Michael Walter, Manuela Bastian, Holger Sven Willenberg, Brigitte Vollmar, Ernst Klar, Ursula Seidler, Georg Lamprecht, Maria Witte

**Affiliations:** 1 Division of Gastroenterology, Department of Medicine II, Rostock University Medical Center, Ernst-Heydemann-Str. 6, 18057 Rostock, Germany; 2 Institute for Biostatistics and Informatics in Medicine and Ageing Research, Rostock University Medical Center, Ernst-Heydemann-Str. 8, 18057 Rostock, Germany; 3 Institute for Clinical Chemistry and Laboratory Medicine, Rostock University Medical Center, Ernst-Heydemann-Str. 6, 18057 Rostock, Germany; 4 Division of Endocrinology and Metabolism, Department of Medicine II, Rostock University Medical Center, Ernst-Heydemann-Str. 6, 18057 Rostock, Germany; 5 Institute of Experimental Surgery, Rostock University Medical Center, Schillingallee 69a, 18057 Rostock, Germany; 6 Department of General, Thoracic, Vascular and Transplantation Surgery, Rostock University Medical Center, Schillingallee 35, 18057 Rostock, Germany; 7 Department of Gastroenterology, Hepatology and Endocrinology, Hannover Medical School, Carl-Neuberg-Str. 1, 30625 Hannover, Germany

**Keywords:** Ileocecal resection, Mice, Short bowel syndrome, Intestinal failure, Clinical outcome, Intestinal adaptation

## Abstract

**Background:**

Short bowel syndrome results from extensive small bowel resection and induces adaptation of the remaining intestine. Ileocecal resection (ICR) is the most frequent situation in humans. Villus hypertrophy is one hallmark of mucosal adaptation, but the functional mechanisms of mucosal adaptation are incompletely understood.

**Aims:**

The aim of the study was to characterize a clinically relevant model of short bowel syndrome but not intestinal failure in mice and to identify outcome predictors and mechanisms of adaptation.

**Methods:**

Male C57BL6/J mice underwent 40% ICR and were followed for 7 or 14 days. Small bowel transection served as control. All mice underwent autopsy. Survival, body weight, wellness score, stool water content, plasma aldosterone concentrations, and paracellular permeability were recorded.

**Results:**

Unlike controls, resected mice developed significant diarrhea with increased stool water. This was accompanied by sustained weight loss throughout follow-up. Villus length increased but did not correlate positively with adaptation. Plasma aldosterone concentrations correlated inversely with body weight at day 14. After ICR, intestinal epithelial (i.e., tight junctional) sodium permeability was increased.

**Conclusions:**

40% ICR results in moderate to severe short bowel syndrome. Successful adaptation to the short bowel situation involves villus elongation but does not correlate with the degree of villus elongation alone. In addition, increased intestinal epithelial sodium permeability facilitates sodium-coupled solute transport. Hyperaldosteronism correlates with the severity of weight loss, indicates volume depletion, and counterregulates water loss.

**Electronic supplementary material:**

The online version of this article (10.1007/s10620-018-5420-x) contains supplementary material, which is available to authorized users.

## Introduction

Intestinal failure is a severe condition with poor prognosis and occurs when the intestine cannot provide sufficient caloric and fluid absorption [1]. Short bowel is one major reason of intestinal failure. Mesenteric ischemia and repetitive surgical resections such as in Crohn’s disease as well as previously failed surgery are the main conditions leading to short bowel, demonstrating that patients with short bowel syndrome (SBS) constitute a very heterogeneous population [2–4]. Adaptation of the intestine is a slow process that is mainly determined by the length and the specific segments of the remnant small bowel and the presence or absence of the ileocecal valve. While adaptation may eventually result in oral autonomy in some patients, temporary or permanent parenteral nutrition and surgical interventions such as reconstructive surgery, lengthening procedures, or—in selected cases—intestinal transplantation are the treatment options for intestinal failure [5, 6]. Additionally, nutritional approaches as well as hormone and growth factor stimulation are strategies to enhance intestinal adaptation in SBS patients [7]. Teduglutide, a GLP-2 analogue, has recently been introduced into clinical practice and has been shown to reduce fluid requirements of parenteral support [8]. Since treatment options are limited in SBS, understanding the mechanisms of adaptation and developing new drugs are both crucial. Genetic risk factors for SBS and severe outcomes have been identified [9, 10]. However, mechanistic studies in humans are difficult to perform due to the heterogeneous patient population. Therefore, a short bowel model in animals that mimics severe SBS in humans is warranted. Such a model should include extensive distal intestinal resection because this is the most common resection leading to SBS in humans [5]. An end jejunostomy is not applicable in rodents. Mice present a unique opportunity to study specific mechanisms by using genetically altered knockout models. The first experimental data on short bowel in mice have been reported by Helmrath et al. [11]. These authors demonstrated feasibility of a 50% resection of proximal small bowel with an end-to-end anastomosis. Increasing the resected segment to 75% leads to a significant decrease in survival without enhanced adaptation [11, 12].

In this study, we performed a 40% ileocecal resection (ICR) originally described by Dekaney et al. [13], leading to an orally compensated SBS in mice. We show that 40% ICR induces severe short bowel syndrome with sustained stool water and body weight losses, which are counteracted by hyperaldosteronism. In this SBS model, increased intestinal epithelial sodium permeability is a mechanism of epithelial adaptation that is active in addition to villus hypertrophy. This may allow back-leakage of sodium to sustain increased transcellular sodium-coupled transport of nutrients.

## Methods

### Mice

C57BL6/J mice were purchased from Jackson Laboratory (Bar Harbor, USA) and bred in the animal facilities at the Institute for Experimental Surgery, University Medical Center Rostock. Mice were housed in groups of up to five under conventional conditions with free access to standard chow and water. All mice were switched to liquid diet (AIN 93G, ssniff, Soest, Germany) 2 days before surgery and maintained on this diet throughout the experiment. Male mice at the age of 3–6 months weighing 29 ± 2 g were randomly assigned to either ileocecal resection (ICR, *n* = 80) or sham operation (Sham, *n* = 40). 24 ICR and 15 sham mice were operated with the intention to survive 7 days (POD 7). Another 56 ICR and 25 sham mice were operated with the intention to survive 14 days (POD 14).

### Operative Procedure

Mice were weighted and anesthetized by i. p. injection of ketamine (100 mg/kg bw) and xylazine (15 mg/kg bw). To prevent intraoperative hypoxemia, mice were orally intubated with a 22-gauge cannula and ventilated mechanically with room air (200 µl and 120 breaths/min) using a mouse ventilator (Minivent 845, Hugo Sachs Elektronik-Harvard Apparatus GmbH, March-Hugstetten, Germany). Mice were placed on a heating plate, and body temperature was continuously monitored by rectal probe. The procedure was performed under semi-sterile conditions using an operating microscope with 16× magnification for the anastomosis (Universal S3, Zeiss, Germany). The operative technique of ICR was adapted from Dekaney et al. [13]. After midline incision, the ileocecal junction and the ileum were eviscerated. The mesentery of the bowel segment was exposed, incised, and ligated using microclips (Weck^®^, Horizon™, Teleflex Medical, Kernen, Germany). Then, the bowel was transected 12 cm proximal to the ileocecal junction and immediately distal to the cecum in the ascending colon. The resected bowel was removed, and the length of the specimen was measured. Intestinal continuity was restored by an end-to-end, full-thickness jejuno-colonic anastomosis by performing 11–13 interrupted stitches and using a 10-0 monofilament suture (Ethilon, Ethicon, Norderstedt, Germany). The bowel was replaced into the abdominal cavity which was irrigated with 1 ml saline. The abdomen was closed in 2 layers using 6-0 Maxon. Sham control mice received a single transection 12 cm proximal to the cecum with end-to-end anastomosis in the same fashion without resection but also with temporal evisceration of the bowel. Immediately after extubation, all mice were weighted, volume resuscitated with 1 ml saline subcutaneously, and received 5 mg/kg bw carprofen as an analgesic. All surgical procedures were performed by the same surgeon (MW). Mice were allowed to recover in a heated terrarium (29 °C) for approximately 4 h and then returned to individual cages with free access to liquid food and water.

### Clinical Examination

The animal wellness score, as summarized in Table 1 [14], was measured daily to assess the general condition of mice. Body weight was also recorded daily. Postoperative weight loss was normalized to the preoperative weight.

**Table 1:** Wellness score modified according to Komen et al. [14]

Parameter	Grading	Score
Activity	Normal/medium/low	2/1/0
Fur	Smooth/fluffy/erect	2/1/0
Eyes	Clean open/clean closed/dirty closed	2/1/0
Able to stand strait	Yes/no	1/0
Posture	Normal/modestly curled/fully curled up	2/1/0
Position on feet	Normal/high	1/0
Shivering	Yes/no	0/1

### Measurement of Stool Water Content

In a random group of ICR (*n* = 13) and sham-operated mice (*n* = 3), stool water content was measured at the day of surgery (0 h) and 2d, 5d, 7d, 10d, and 14d after surgery as previously described [15]. First, mice were individually placed in empty cages. Fresh stool was collected in pre-weighed tubes [e] and immediately closed. After weighing again [w], tubes with fresh stool were opened and placed in a hot air oven at 85 °C. After 24 h, the tube was once again weighed [d] and stool water content (%) was calculated as follows:

$$\left( {w - d} \right)/\left( {w - e} \right) \times 100$$

### Autopsy Examination

Autopsies were performed in all mice as far as possible to identify complications. The reasons for death were classified as follows: ileus with obstruction (stenosis of the anastomosis or adhesion formation) or ileus without obstruction (massive dilatation in the absence of stenosis or adhesion formation), abdominal sepsis (insufficiency of the anastomosis, presence of abdominal fibrin coating or abscesses) and others (such as bleeding or rupture of the abdominal wall).

### Histological Examination

Paraffin-embedded, hematoxylin-, and eosin-stained tissue slides were used for morphometric analysis of intestinal adaptation. In a blinded manner, 5 well-oriented full-length villi per sample were measured and averaged using Axio Observer inverted microscope (Zeiss) and Zen 2.3 software.

### Humane Endpoints

A wellness score below 4 points, body weight loss of more than 20% from the initial weight, a distended abdomen together with persistent constipation, or dehydration combined with massive diarrhea were defined as humane endpoints. Mice that reached one or more humane endpoints were killed immediately and underwent autopsy.

Mortality was classified in early (occurring between postoperative days 0–3), intermediate (between days 4–7), and late mortality (between days 8–14).

### Measurement of Plasma Aldosterone Level

In a random group of 9 ICR mice, EDTA blood was collected at day 14. The MassChrom^®^ Steroid Panel (Chromsystems Instruments & Chemicals GmbH, Gräfelfing, Germany) was run on 500-µl plasma samples. Solid-phase extraction was carried out according to the manufacturer’s protocol. An Agilent 1290 UPLC series binary pump was used with Chromosystems analytical columns (Order No. 72110). Agilent MassHunter Quantitative Data Analysis Software (B.07.01) was used for data analysis. A 1/*x*^2^ weighting factor was applied during linear regression of the calibration curves. The quantitation using MassHunter Quantitative Software was performed by comparing chromatographic peak area ratio to a fixed concentration of the internal standard.

### Ussing Chamber Studies

Mouse jejunum was obtained and for 10 min preincubated in ice-cold recording buffer containing 1 µM indomethacin. The tissue was then freed from the seromuscular layer under a dissecting microscope and analyzed in Ussing chambers with 5 ml half cell volume and 0.24 cm^2^ surface area with AgCl electrodes (Karl Mussler, Scientific Instruments). Recording buffer contained 115 mM NaCl, 0.4 mM Na_2_HPO_4_, 2.4 mM NaH_2_PO_4_, 5 mM KCl, 1.2 mM MgCl_2_, 1.2 mM CaCl_2_, 25 mM NaHCO_3,_ and 5 mM Glucose and pH 7.4 and was continuously gassed with 95% O_2_ and 5% CO_2_ at 37 °C.

Mucosal to serosal FITC 4-kDa dextran flux was measured by adding it at 40 ng/ml into the mucosal compartment. Fluorescence intensity increase in the serosal chamber was measured after 1 and 2 h using a plate reader. Dextran flux was calculated from a simultaneously determined standard curve. Na/Cl dilution potentials were recorded after replacing 57.5 mM NaCl with 115 mM Mannitol in the apical and basolateral buffer. PNa^+^/PCl^−^ was calculated applying the Goldman–Hodgkin–Katz equation [16].

### Statistics

A prospective database was established to monitor animal data, technical aspects, outcome, and complications using Microsoft Access 2010. All data were analyzed using GraphPad Prism 7.0 (GraphPad Software CA, USA). Data are presented as mean ± standard error of the mean (SEM) for the indicated number of mice (n) per group. Kaplan–Meier curves were compared using the log-rank Cox–Mantel test. Correlation between villus height, body weight, and plasma aldosterone at day 14 was assessed by linear Pearson or nonlinear Spearman regression analysis. Group comparisons were performed by the nonparametric Kruskal–Wallis test before subgroups were tested pairwise with Mann–Whitney *U* test. Nonparametric Wilcoxon signed-rank test was used to compare related samples. Significance level 0.05 (or Bonferroni-adjusted *α*_adjust_ as indicated in the figure legends) was considered.

## Results

### Early and Late Survival

24 ICR and 15 sham mice were operated with the intention to survive 7 days (POD 7). Survival in the POD 7 group was 70% in the ICR group and 75% in the sham group. Another 56 ICR and 25 sham mice were operated with the intention to survive 14 days (POD 14). Survival in the POD 14 group was 49% in ICR mice and 75% in sham mice.

**Fig. 1:**
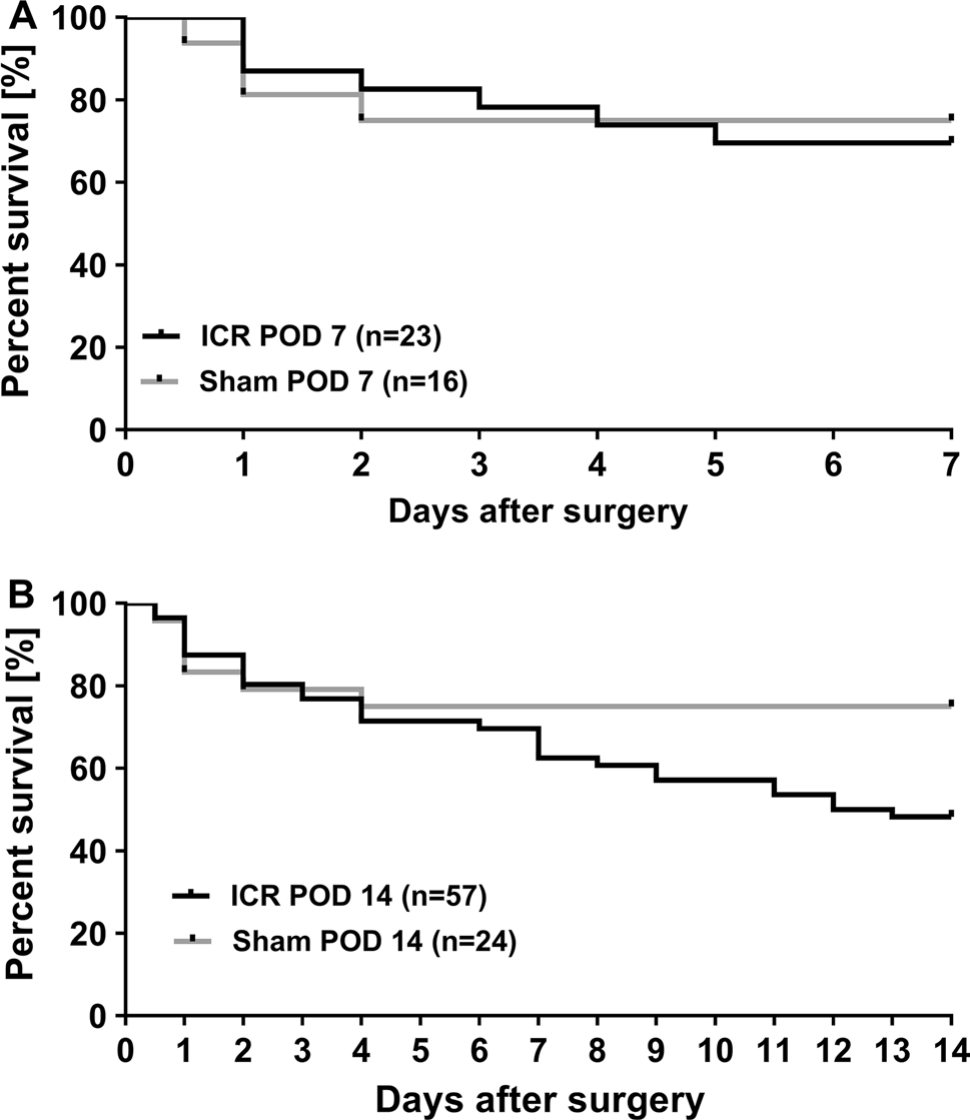
Kaplan–Meier curves of mice who underwent ileocecal resection (ICR) or sham operation with defined experimental endpoint at day 7 (**a**) or day 14 (**b**) (POD). Mice that survived until the expected observation period were defined as “survivor,” whereas mice that died unexpectedly or were killed because of reaching a humane endpoint were defined as “dead.” Similar early survival of ICR and sham controls (POD 7, *p* = 0.813). **b** At later time points (POD 14), survival curves of sham and ICR mice separated although the difference did not become significant during the observation period (*p* = 0.061)

The 7-day survival was comparable between ICR and sham mice (Fig. 1a). Complications occurred predominately early in ICR mice (71% of deaths) and exclusively early in sham mice (100% of deaths). The 14-day survival in ICR was lower compared to sham mice, although this was not statistically significant (*p* = 0.061, Fig. 1b). In the ICR POD 14 group, mortality predominantly occurred early (45%) and to equal rates in the intermediate and late postoperative period (27.5% each). In the POD 14 sham group, mortality occurred mostly early (83%) and only to a small extent (17%) in the intermediate postoperative period.

### Complications

**Fig. 2:**
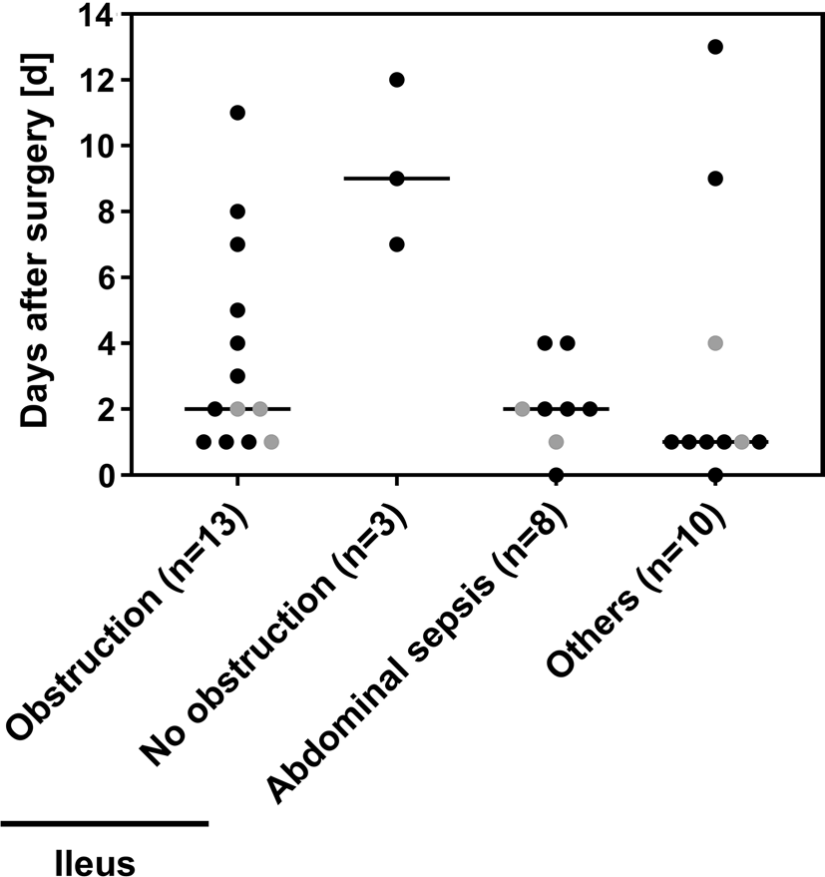
Absolute frequency of complications in ICR and sham mice related to the days of occurrence. Each symbol represents one mouse that died spontaneously or was killed because of reaching a humane endpoint: black, resected mice (*n* = 28); gray symbols indicate sham-operated animals (*n* = 6). Median is shown as a horizontal line

**Fig. 3:**
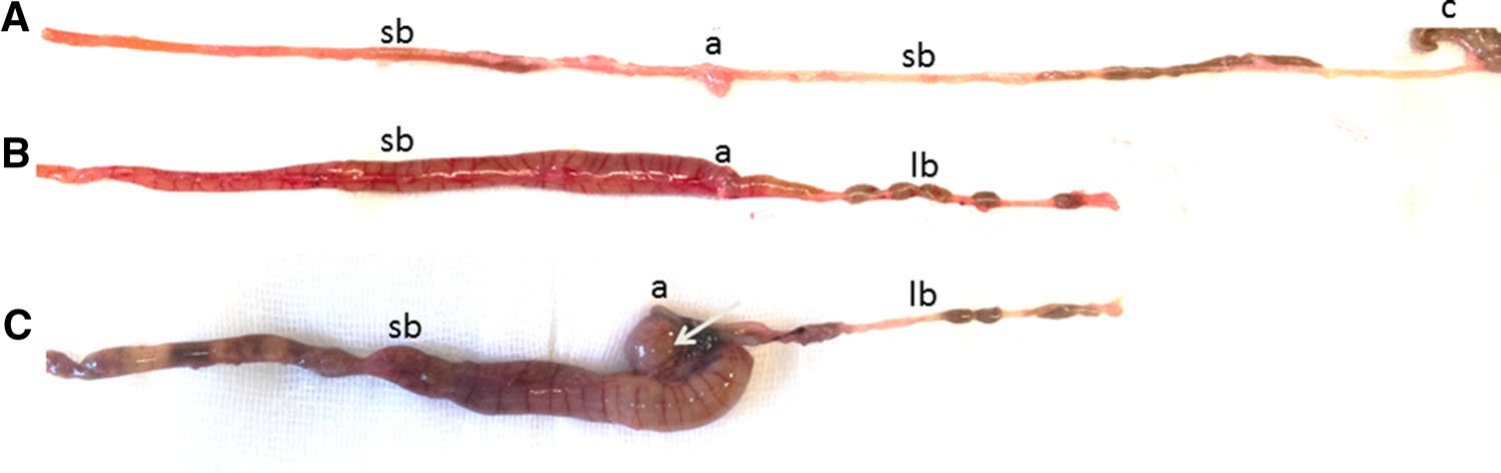
Gross morphology of the intestine after sham surgery (**a**) or ileocecal resection (**b**, **c**). Adapted small bowels displayed some dilatation over the entire length at 14 days after resection (**b**). Mechanical ileus with massive pre-anastomotic dilatation due to obstruction (arrow) at day 11 (**c**). sb: small bowel, lb: large bowel, a: anastomosis, c: cecum

A total of 46 of 120 mice (38%) were killed before the end of the experiment or died spontaneously due to a complication. Figure 2 shows the different complications and their occurrence along the postoperative course for 34 of 46 mice with a discernible complication or cause of death. In the remaining 12 mice, the cause of death remained unclear. The most frequent complication was ileus due to obstruction that occurred between day 1 and day 11 (median day 2) (Figs. 2, 3c.). Within this cohort, stenosis of the anastomosis was seen in 7 of 13 mice. Six mice developed ileus due to other mechanical reasons, e.g., by extensive perianastomotic adhesion formation with kinking of the remaining intestine. Of note, ileus without mechanical reason developed late in 3 of 16 mice (median day 9). Abdominal sepsis was identified in 8 animals at a median of 2 days after surgery. In 6 of these mice, anastomotic insufficiency was identified. Other complications occurred in 10 mice and included gastrointestinal bleeding, rectal prolapse, fascial dehiscence, and bowel ischemia.

### Predictors of Survival

**Fig. 4:**
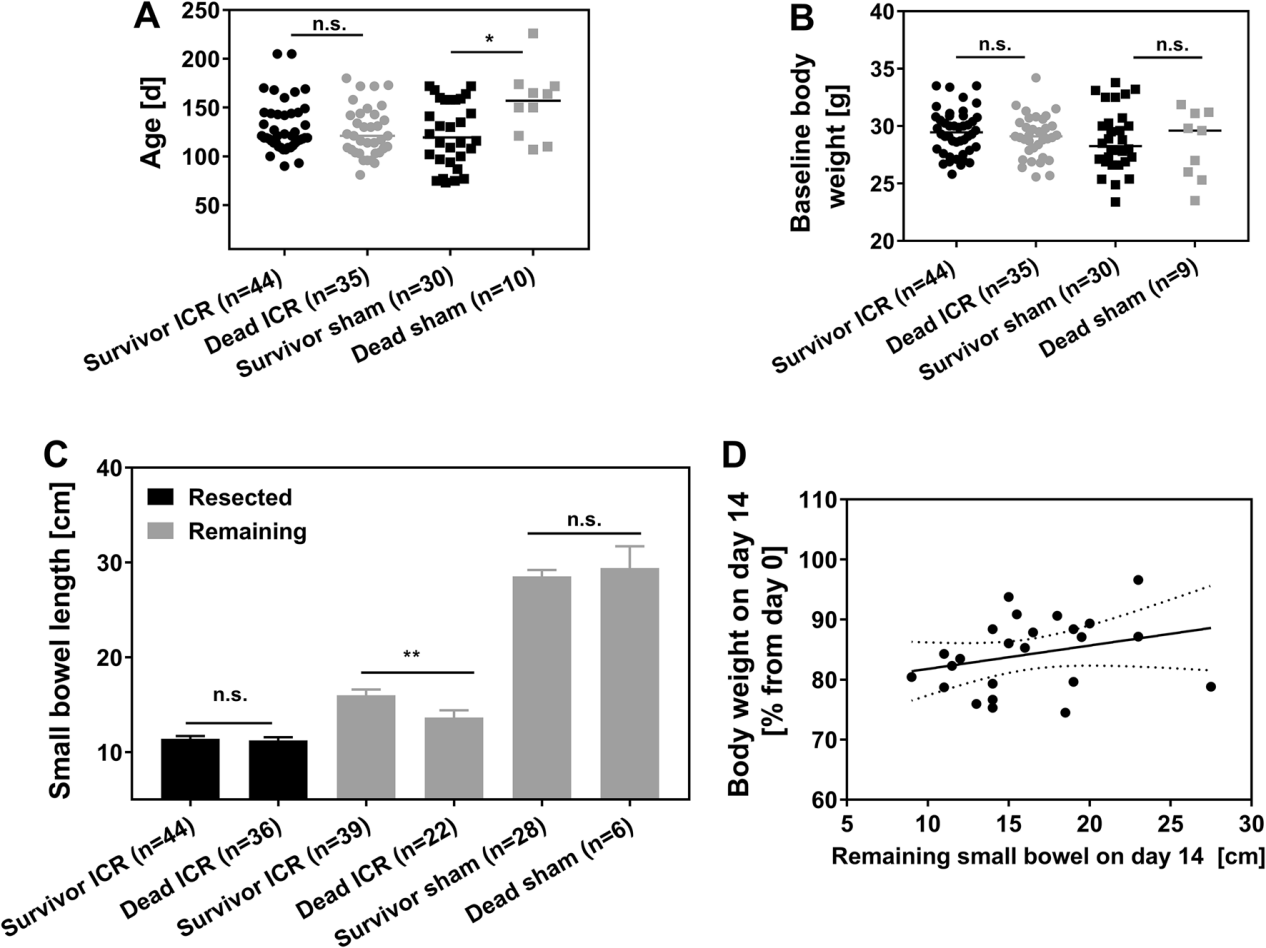
Age (**a**) and baseline body weight (**b**) of resected (ICR) and sham control mice that reached the expected end point (survivor) or died earlier (dead) at time of surgery. Each symbol represents one mouse. Medians are shown as horizontal line. **a** *p = 0.024, *U* test. **c** Small bowel lengths of survivors and dead ICR mice (mean ± SEM, **p = 0.008, *U* test). **d** Correlation between remaining small bowel length and body weight on day 14 as a percentage from day 0 in ICR mice that survived to the expected endpoint. Pearson *r* = 0.28, *p* = 0.18, *n* = 24

The mean age of resected mice that reached the endpoint was 129.7 ± 25.9 days, similar to that of dropped out ICR mice with 125.2 ± 25.6 days. However, the age of sham mice that died early was significantly higher compared to the age of surviving sham mice (153.9 ± 35.6 days vs. 123.0 ± 33.0 days, *p* = 0.024, Fig. 4a). The absolute body weight at the time of surgery (baseline) did not significantly differ between surviving ICR mice and ICR mice with early death (29.5 ± 1.9 g survivor ICR vs. 29.1 ± 1.8 g dead ICR, Fig. 4b). The same was true for sham control mice (28.8 ± 2.7 g survivor sham vs. 28.4 ± 2.9 g dead sham, Fig. 4b). Resection of the proximal ileum had to be adjusted to the perfusion margins during surgery. As a result of this, the length of the resected segment varied between individual mice. There was no statistically significant difference between mice that reached the endpoint and dropouts (11.4 ± 2.0 cm in surviving mice vs. 11.2 ± 2.1 cm in dropouts; *p* = 0.833, Fig. 4c). Total bowel length in all sham mice was 28.7 ± 4 cm. However, the mean remaining small bowel after 14 days was significantly longer in ICR mice that survived to the designated endpoint (16.0 ± 3.8 cm) compared to mice that died earlier (13.7 ± 3.6 cm, *p* = 0.008; Fig. 4c). However, there was no significant correlation between body weight and remaining small bowel length at day 14 in surviving ICR mice (Fig. 4d).

### Weight Course and Wellness

**Fig. 5:**
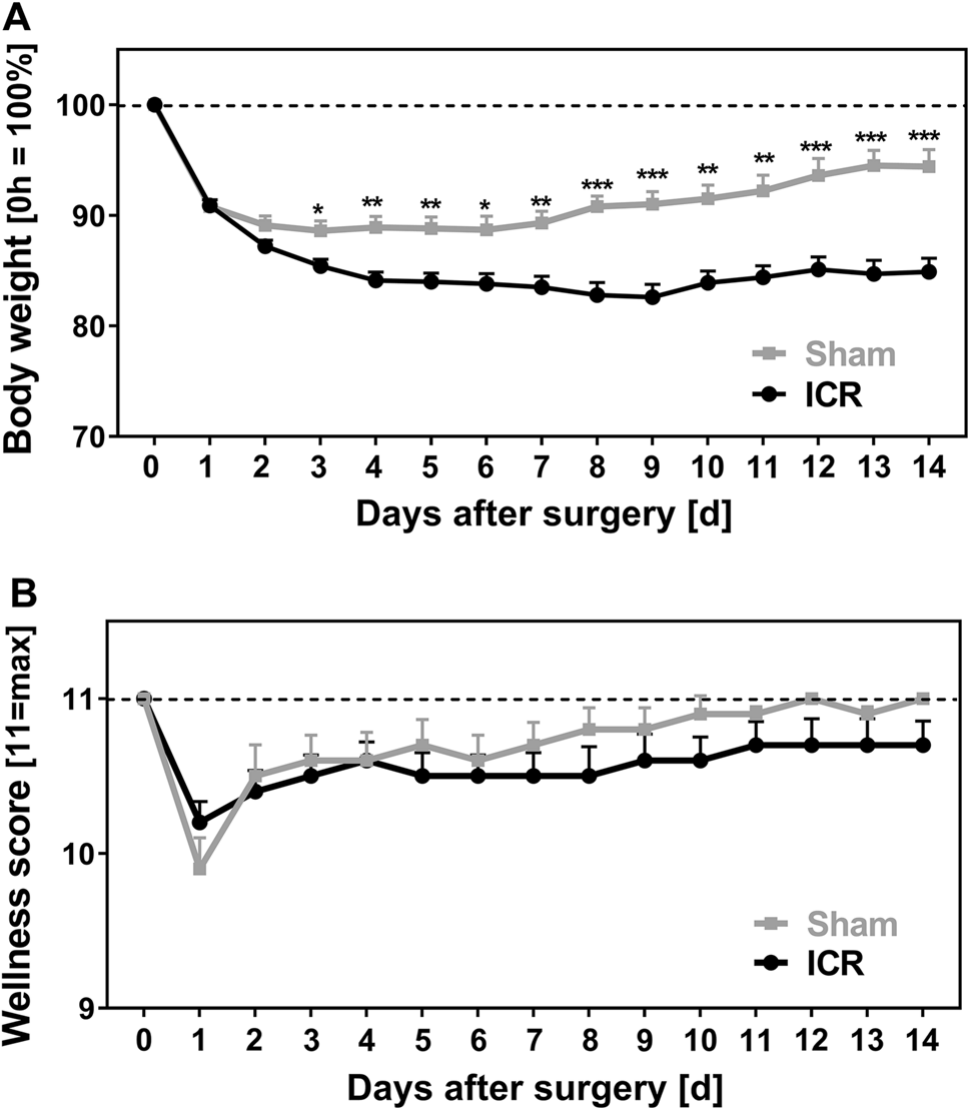
Weight course (**a**) and wellness (**b**) of mice with ileocecal resection (ICR) or sham operation (sham) over time. **a** Body weight as percentage from day 0. **b** Wellness score of 11 indicates maximal wellbeing. Data only from mice that survived until the end of the experiment, those who that died earlier were excluded. All data are shown as mean + SEM. ICR (*n *= 44 d0–d7; *n* = 27–28 d8–d14) versus Sham (*n* = 30 d0–d7; *n* = 17–18 d8–d14) *α*_adjust_ = 0.004; *p < 0.004, **p < 0.001, ***p < 0.0001

To evaluate the clinical outcome after surgery, body weight and wellness score (Table 1) from ICR and sham mice were recorded daily. Figure 5 shows data from mice that reached the intended endpoint. After ICR, mice developed prolonged weight loss. Body weight did not recover but remained almost stable until the end of the experiment and was 84.9 ± 6.3% in ICR mice at day 14. In contrast, sham mice reached their minimal body weight as early as 3 days after surgery (88.6 ± 4.9% from baseline). Unlike resected mice, body weight of sham controls recovered to 94.4 ± 6.4% at day 14. Weight course of ICR mice differed significantly from sham controls from day 3 until day 14 (Fig. 5a).

**Fig. 6:**
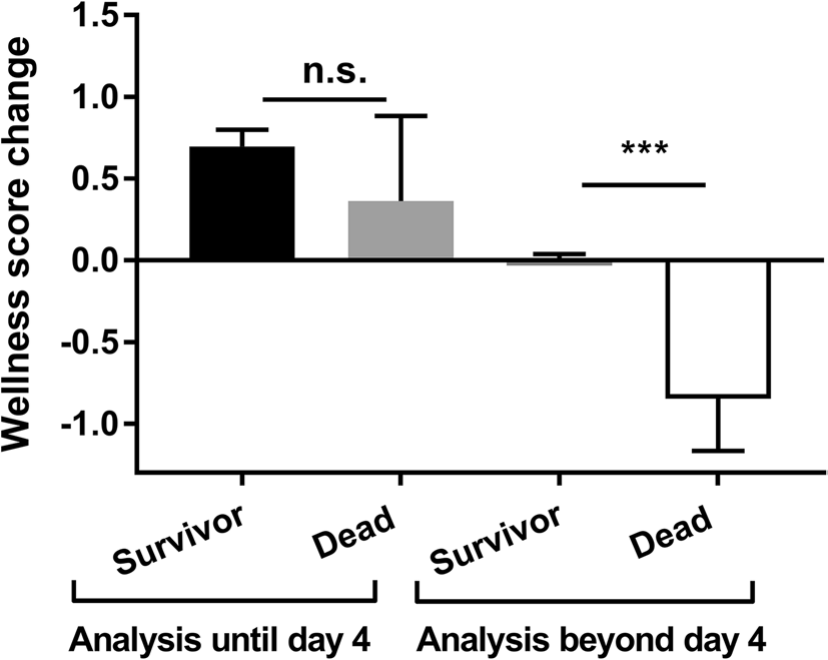
Wellness score change predicts premature death. In surviving mice, the day-to-day wellness score change from 48 h to 24 h prior to visit was calculated for all days. In premature dying mice, the wellness score change from 48 h to 24 h before death was calculated. Mean change during the first 4 days was similar between survivors (*n* = 132, black) and dead mice (*n* = 11, light gray, *p* = 0.827). Thereafter, mean wellness score change for dead mice (*n* = 13, white) was significantly reduced compared to survivors (*n* = 327, dark gray). Mean + SEM, ***p < 0. 001, *U* test

The clinical status of mice after surgery was assessed using the wellness score established by Komen et al. [14] which has a maximum value of 11 (Table 1). Immediately after surgery, mice of both groups showed decreased wellness scores (ICR minimal score 10.2 ± 0.9, sham minimal score 9.9 ± 1.1 both at d1) that recovered almost completely in both groups. Surviving ICR mice did not fare significantly worse than sham controls over the course of the experiment (Fig. 5b). In contrast, mice that dropped out had a lower wellness sore compared to their surviving littermates (not shown). Because animals were taken out of the experiment based on poor status, wellness score was very low by definition on the day of death. In order to find out if the wellness score is predictive of premature death, we calculated *daily changes* of the individual wellness score from 48 to 24 h before the daily visit. Until postoperative day 4, wellness score change was positive both in surviving mice and in mice that died after day 4 reflecting the recovery from surgery and indicating that the wellness score is not predictive of death in the early postoperative period. However, after postoperative day 4, the wellness score declined significantly on the day before death compared to surviving controls (Fig. 6, *p* < 0.001). Thus, a decline in the wellness score after day 4 is an indicator of impending death.

### Stool Water Content

**Fig. 7:**
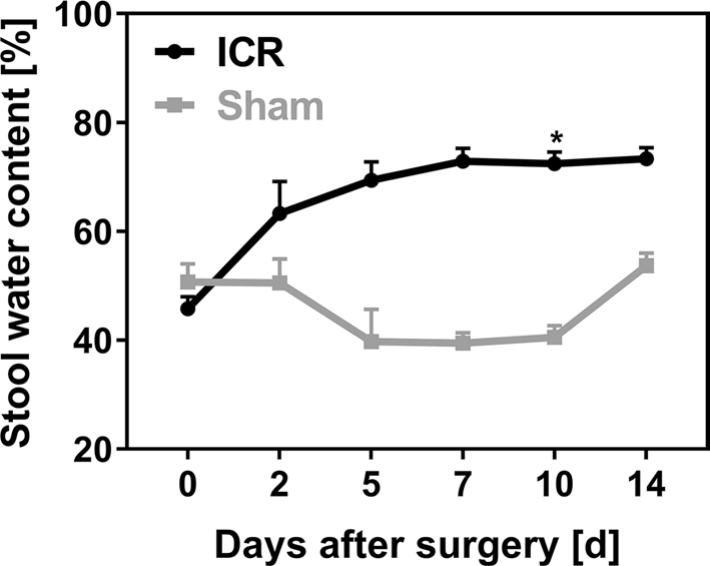
Stool water content was measured before surgery (0 h) and at d2, d5, d7, d10, and d14 after ileocecal resection (ICR, *n* = 10) or sham operation (Sham, *n* = 3) in a separate group. Resected mice had increased stool water contents compared to controls. Mice that died before day 14 were excluded. Data are shown as mean + SEM. *α*_adjust_ < 0.01; *p = 0.007 d10

After ICR, mice developed pasty stools and to a lesser extent diarrhea (score 0) that returned to visually solid stools at the end of the second week after surgery. To quantify diarrhea, we measured stool water content in a random group of 13 ICR and 3 sham-operated mice. ICR mice that reached the endpoint had significantly higher stool water content compared to sham controls (*p* = 0.007, d10). Stool water content did not recover at the end of the observation period in ICR mice (Fig. 7).

### Plasma Aldosterone

**Fig. 8:**
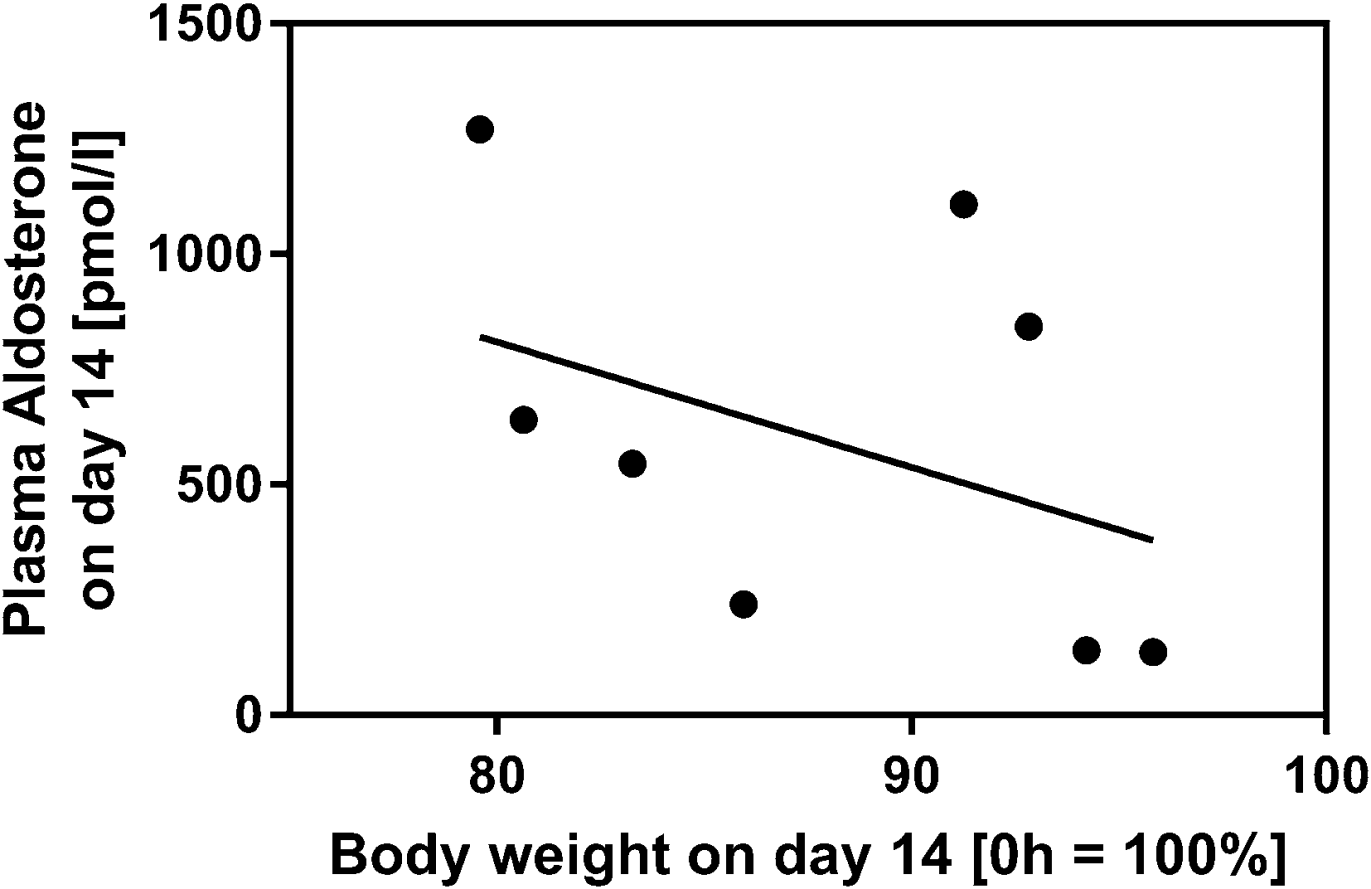
Correlation between body weight and plasma aldosterone concentration. In a random group (*n* = 9) of ICR mice, plasma aldosterone concentration at day 14 was correlated with relative body weight at day 14 (compared to d0). Plasma aldosterone concentration at day 14 was inversely correlated with body weight at d14. Spearman *r* = − 0.6, *p* < 0.05

Mice developed significant water losses through watery stools. However, we did not measure thirst and drinking behavior as compensatory mechanisms to volume depletion because metabolic cages are difficult to maintain on a routine basis. Instead, we measured plasma aldosterone concentrations as an indicator for salt and volume status in a random set of ICR animals and correlated these values with body weight on day 14. There was a significant, inverse correlation of aldosterone levels with increasing body weight (Spearman *r* = − 0.6, Fig. 8). Given that body weight after 14 days is an indicator for adaptation and nutrition status, this suggests that worse adapted mice also have an impaired volume status.

### Histomorphological Adaptation

**Fig. 9:**
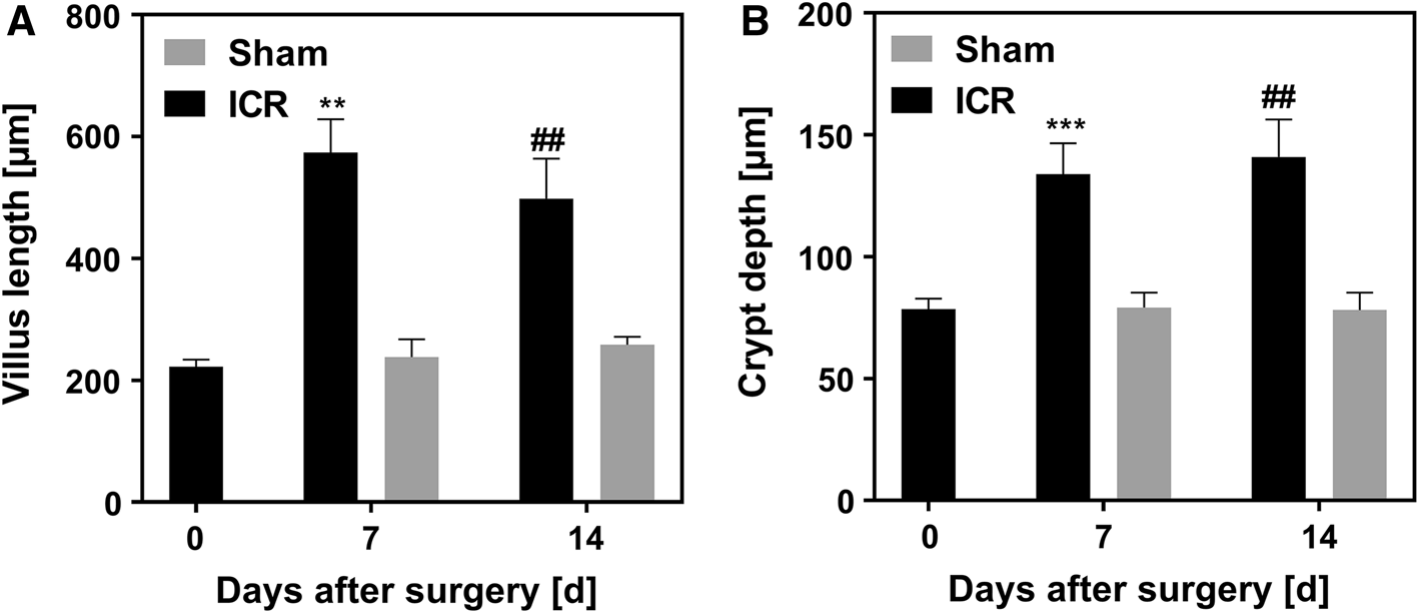
Villus lengths (**a**) and crypt depths (**b**) at time of surgery (0 days), 7 days (*n* = 6 ICR, *n* = 7 sham), or 14 days (*n* = 9 ICR, *n* = 4 sham) after ileocecal resection (ICR) or sham surgery. Data are shown as mean + SEM. **a** **p = 0.0014 ICR 0d versus ICR 7d, *n* = 6 and **##***p* = 0.0011 ICR 0d versus ICR 14d, *n* = 9 paired *t* test. **b** ***p = 0.001 ICR 0d versus ICR 7d, *n* = 6 and ##*p* = 0.0013 0d ICR versus 14d ICR, *n* = 9

As a result of morphological adaptation, resected mice had 2–2.5-fold increased villus heights (Fig. 9a) and crypt depths (Fig. 9b) 7 and 14 days after surgery compared to baseline. Villus lengths and crypt depths of sham-operated mice were not altered after surgery (Fig. 9).

**Fig. 10:**
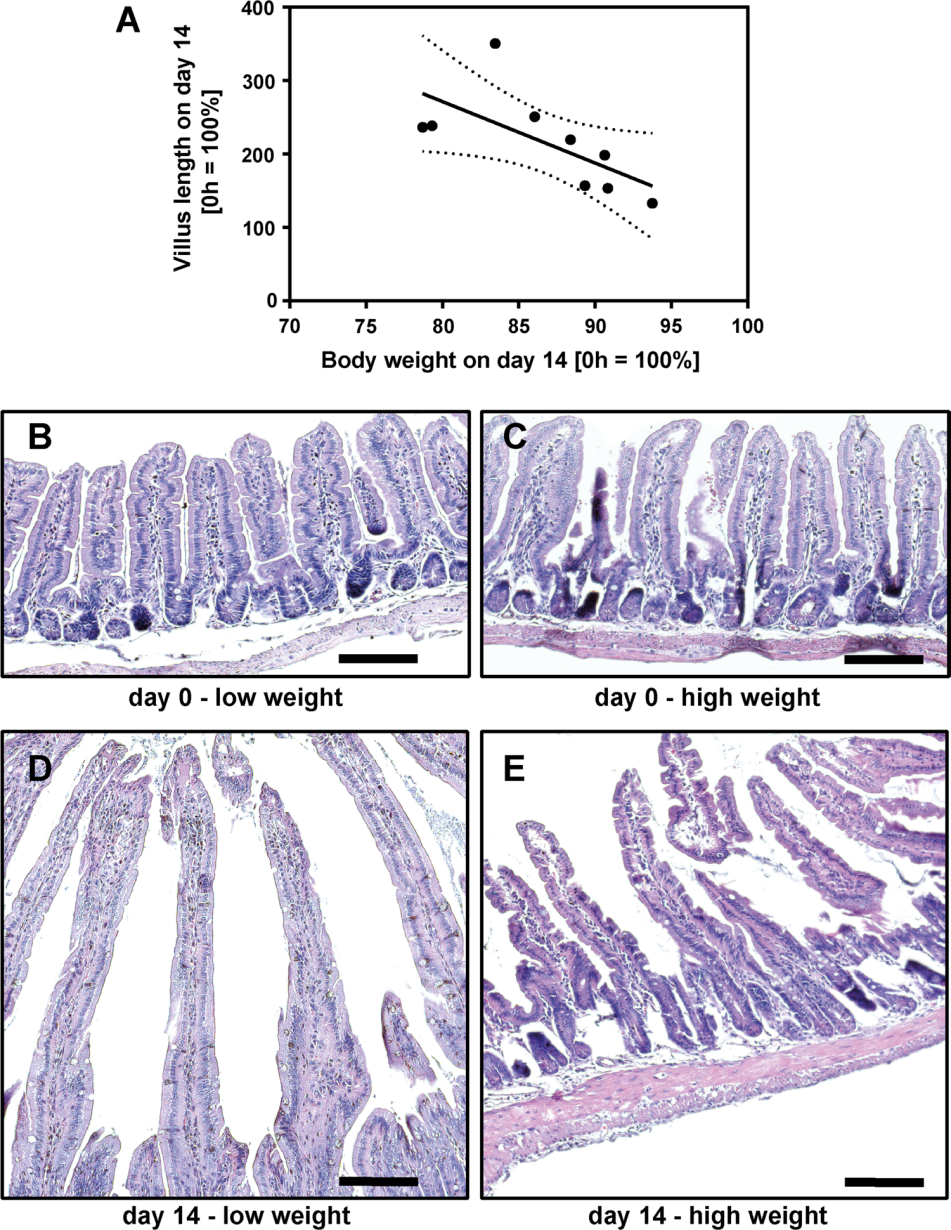
Correlation between body weight and villus length. In a random group (*n* = 9) of ICR mice, relative villus length (compared to d0) was correlated with relative body weight on d14 (compared to d0). Relative villus length was inversely correlated with body weight on d14. Pearson *r* = − 0.67, *p* = 0.05 (**a**). Representative H and E images of resected segments at day 0 (**b**, **c**) and of the corresponding adapted intestines from mice with low (**d**) or high (**e**) body weight at day 14. Note that not only villi become longer but also that the subepithelial and intravillous tissue enlarges. Scale bar represents 100 µm

In mice that survived to day 14, body weight was variable with relative body weight of 74% to 98% of the initial weight (Fig. 4d). While all mice displayed some degree of villus elongation, the increase in villus length on day 14 also had considerable variation. In order to find out if villus elongation is associated with the outcome, we correlated relative villus height with relative body weight in a random group of 9 surviving mice. Interestingly, villus height was inversely correlated with body weight (Pearson *r* = −0.67, *p* = 0.049, Fig. 10). Thus, villus length is not reflected by good clinical adaptation in surviving mice.

### Epithelial Barrier Function

**Fig. 11:**
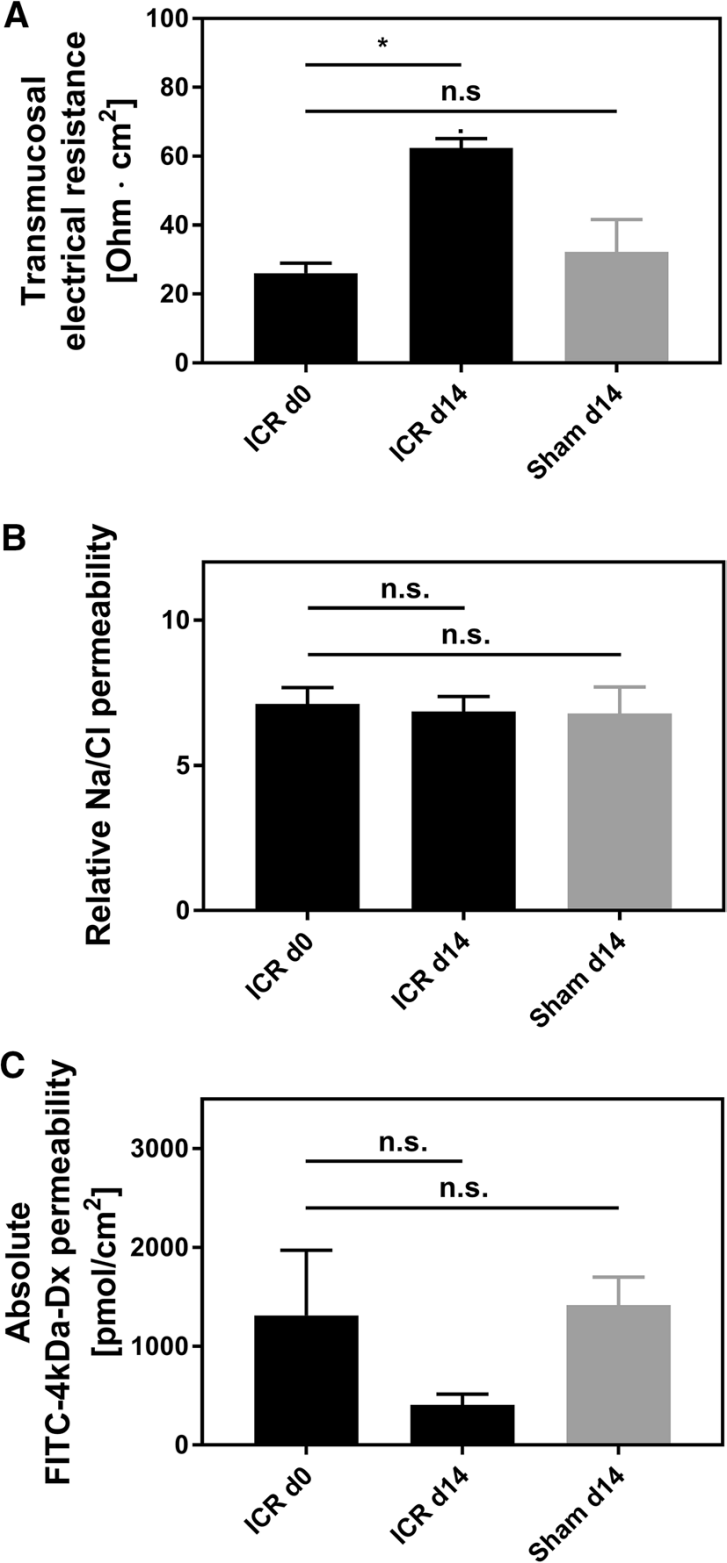
Ussing chamber studies of mucosal barrier function in the jejunum after ileocecal resection. Jejunal tissues were stripped off the serosa and the muscularis. **a** Transmucosal electrical resistance (TMER) did not change in sham-operated mice (*n* = 4). At day 14, the jejunum of ICR mice displayed significantly higher TMER (*n* = 9; *p* < 0.05, Wilcoxon test). **b** Relative Na/Cl permeability, which is represented by epithelial tight junctions, was calculated from Na/Cl dilution potentials. Both after sham and after ICR, ion selectivity was unchanged (sham *n* = 6; ICR *n* = 9; *p* > 0.05, respectively) despite villus hypertrophy, which only occurred in ICR animals. **c** The paracellular leak pathway was studied by 4 kDa fluorescein isothiocyanate–dextran FITC 4-kDa dextran diffusion. At day 14, the permeability in sham-operated mice (*n* = 7) was not significantly changed. At day 14, permeability was numerically lower in ICR mice, although not statistically significant (*n* = 6; *p* = 0.16, Wilcoxon test)

Villus elongation alone did not indicate good adaptation. Thus, we studied epithelial barrier function in the Ussing chamber. In sham controls, both leak and pore pathway indicators were indistinguishable from day 0 (Fig. 11a–c). To address the pore pathway, we first measured transmucosal electrical resistance (TMER). After 14 days, TMER was increased more than twofold in the ICR animals (Fig. 11a). However, because after 14 days, villus length also grew more than twofold, electrical resistance of the subepithelium interferes with TMER. In order to address epithelial permeability directly, we therefore assayed the epithelium specific sodium/chloride permeability ratio using diffusion potential measurements. No significant difference was detectable after 14 days (Fig. 11b). Because the subepithelial permeability is not ion selective, this indicates an increased sodium permeability at the epithelial layer, i.e., at the tight junction. In line with this, the transmucosal leak pathway, reflected by 4 kDa FITC-dextran flux, was not significantly impaired (Fig. 11c), again indicating changes on the epithelial level.

## Discussion

Several small animal models to study intestinal failure have been established over the last 50 years [13, 15, 17–21]. While early studies have focused on animals such as rats [17–19] or rabbits [20], murine models have recently gained more attention [22]. This is largely due to the availability of genetically modified mice with the opportunity to study the role of specific genes in knockout animals. In addition, the postoperative functional anatomy should mimic the situation in human patients. Moreover, the model should be severe enough to induce SBS but must not be too severe in order not to induce intestinal failure which would require parenteral support. Thus, to study mechanisms of intestinal adaptation and genetic risk factors under SBS conditions, we have analyzed a SBS model by performing 40% ICR in adult C57BL/6 mice. We gathered data from 120 mice over 7 or 14 days after surgery and focused on clinical outcome (weight course, wellness, stool water) and related mechanisms (plasma aldosterone and barrier function), as well as perioperative complications and late outcome.

### Technical Aspects

So far, most animal models have used proximal small bowel resections [18, 19, 21]. However, while these models are well suited to study mucosal remodeling, anatomically they do not resemble the most common form of human SBS [21]. An extensive resection of the distal ileum and the proximal colon is the most frequent anatomic situation in human SBS [5, 23]. Because the cecum has a high biological relevance in rodents, ICR conceivably results in even more severe disease than small bowel resection only [24]. On the other hand, increasing the extent of the resection results in severe clinical outcome and 100% mortality beyond POD 29, as recently described by Matsumoto in a model of 75% ICR [25]. Moreover, extension of the resection to more than 50% does not enhance the adaptive response of the intestine as shown in a model of 75% versus 50% proximal small bowel resection, suggesting that a more extensive resection will not provide additional insight into the mechanisms of adaptation [12]. In order to model the most frequent type of human SBS, we thus performed 40% resection of the distal small intestine and the cecum to induce short bowel conditions in mice.

When studied for 7 days, mortality in this model was between 25 and 30% both in sham and in ICR mice. Because early mortality in both groups was related predominantly to surgical complications, intestinal failure with an insufficient adaptive response apparently did not occur at this time. Early death suggests that anesthesiologic and perioperative management influences the outcome. To this end, four specifics in the perioperative management were found to be important in our hands. (1) Both intraperitoneal ketamine/xylazine and isoflurane anesthesia are established options. Mice recover more quickly after isoflurane anesthesia. However, the bowel dilates and makes suturing more complicated. Thus, we used intraperitoneal ketamine/xylazine. (2) To ensure stable oxygenation during surgery, mice were routinely intubated. However, sometimes multiple intubation attempts were necessary and may then even have caused trauma. (3) Postoperatively mice were volume resuscitated with 1 ml saline subcutaneously and received 5 mg/kg carprofen as an analgesic. (4) Unlike others, we did not apply antibiotics perioperatively because single shot antibiotics may influence postoperative outcome with regard to infections but may also influence the intestinal microbiome and thus may interfere with adaptation to the SBS situation [26]. Given the fact that we detected only two cases of peritonitis at autopsy, it appears suitable and reasonable to omit antibiotics.

We also modified surgical details during the establishment of this model. We used 10-0 suture instead of 9-0 suture as described by Dekaney and by Helmrath [13, 26], and we applied 1 ml pre-warmed saline into the abdominal cavity at the end of the procedure in order to prevent adhesions. In line with other rodent resection models, we switched mice to a liquid diet 2 days prior to surgery and maintained them on this diet until the end of the experiment. Nonetheless, 10% of the mice still died from intestinal obstruction. Thus, maintaining mice on a liquid diet was not efficient to prevent intestinal obstruction completely. In control experiments, water content was not increased in the stool of mice that were fed liquid diet compared to stool of mice that were fed solid pellets 2 days before surgery (data not shown). In line with this, stool water content of sham mice was also not increased compared to baseline, indicating that the increase in stool water was specific for short bowel conditions.

In contrast to other studies, we operated male C57BL6/J mice 3–6 months of age instead of 2–3 months as described for an ICR model and for a proximal small bowel resection [13, 26]. However, our study revealed no significant correlation between the incidence of complications and the age of mice at the time of surgery.

### Outcome and Predictors of Outcome

With regard to early postoperative survival, our data agree with earlier studies. Helmrath et al. [11] as well as Rigby et al. [27] also found that most mice die within the first 3 days. Few publications describe survival beyond day 14 after ICR [13, 25]. Dekaney et al. published an excellent 91% survival after 16 weeks in a 40% resection model with few mice dying early postoperatively [13]. None of these publications reported autopsy findings, which would allow insight into surgical complications and mechanisms of decompensation of the short bowel situation after extensive ICR.

One dreaded complication after extensive bowel resection in humans is anastomotic insufficiency. We detected insufficiency in only 5% of all anastomoses. A more significant problem in mouse abdominal surgery is extensive adhesion formation. In order to avoid this, we flushed the abdomen with saline at the end of the operation, but this did not completely prevent formation of adhesions. In addition, we fed mice a liquid diet throughout the entire experimental phase to prevent stool impaction at the anastomosis [11]. Despite these measures, ileus was the most common complication in our series. In the first week, ileus was exclusively due to obstruction. At autopsy, stenosis of the anastomosis was the most common cause for obstruction. Also, severe perianastomotic adhesions with kinking or a funnel-like luminal appearance indicated obstructive ileus. Ileus was also the predominant complication after day 7. However, in some mice that died from late ileus, obstruction was not detected at autopsy. Remarkably, these mice displayed massive dilatation of the remaining small intestine without a downstream obstructive segment. Of note, prior to death, these mice had developed severe weight loss, sustained severe diarrhea, and excessively high plasma aldosterone concentrations two orders of magnitude higher than the normal plasma concentration (data not shown). It is likely that in late ileus, paralysis occurred secondarily due to volume depletion and electrolyte disturbances. In these mice, SBS may have been compensated initially after the resection, but later adaptation may have been insufficient to sustain clinical recovery. In line with this, we observed an inverse correlation between plasma aldosterone concentration and body weight of resected mice that survived until day 14. This suggests that 40% ICR is a model of SBS-induced weight loss and volume depletion and is well suited to study moderate to severe SBS—but not intestinal failure because it is severe enough to stimulate adaptation as well as mild enough to allow survival of most mice without parenteral support.

The wellness score adapted from Komen et al. [14] was originally described for a mouse model of colorectal anastomotic leakage. Our study showed that it is suitable to detect perioperative complications. In addition, we found that after day 4 any decline in the wellness score predicted death or a humane endpoint within 24 h. In contrast, ICR mice that reached the intended endpoint at day 14 displayed nearly normal wellness scores that were not different from sham controls again indicating compensated adaptation to the SBS situation. Thus, in the ICR model of SBS, the wellness score is a good predictive marker for decompensation toward intestinal insufficiency and early death.

Neither age nor baseline body weight were predictive for early death of ICR mice in this model. Age but not body weight of sham control mice that died early was significantly higher compared to sham mice that reached the expected endpoint. More importantly the age of surviving sham mice was not different from ICR mice, indicating that the intended interventional group was well matched for age.

### Pathophysiology of Adaptation in the ICR SBS Model

The length of the remaining small bowel after 14 days was significantly increased in ICR mice that survived to the designated endpoint compared to mice that died earlier. But correlation of the length of the resected segment at the time of the operation and the length of the remaining small intestine at autopsy did not indicate longitudinal growth as a mechanism of adaptation (data not shown). This is in line with previous findings by Dekaney et al. [13]. Furthermore, we correlated body weights of surviving mice at day 14 with their remaining bowel lengths, in order to find out if differences in body weight reflect a different severity of SBS, but again there was no correlation. Thus, in surviving mice, the variation in remaining bowel lengths was not responsible for the severity of SBS.

ICR mice displayed persistent weight loss that was significantly higher compared to sham controls. In line with the observations of Dekaney et al. [13], neither body weights of ICR mice nor body weights of sham controls fully recovered until day 14 after surgery. Also in line with the study of Dekaney et al. [13], villus lengths and crypt depths were 2–2.5-fold higher in ICR mice at days 7 and 14 compared to baseline. In contrast, in our adult mice histomorphological adaptation was almost complete after 7 days. However, ICR mice showed a high degree of variation both in body weight and in villus length on day 14. Unexpectedly, the increase in villus length was inversely correlated with the relative body weight in mice that survived until the end of the experiment. Thus, good clinical adaptation as assessed by recovery of body weight does not require extensive villus elongation. This finding suggests that other epithelial mechanisms, such as barrier function and transport, are also operative during adaptation. In order to find out if epithelial barrier function is involved in adaptation, we studied the two major paracellular routes, the leak, and the pore pathway [28]. While the epithelial tight junction barrier is the main determinant of paracellular transport in vivo, the subepithelial and intravillous tissue, which cannot be removed by stripping, contributes to the transmural barrier that is being measured in the Ussing chamber in vitro [29]. This was reflected by the transmucosal electrical resistance (TMER) which was increased in a similar magnitude as the villus length and the subepithelial intravillous tissue. Similarly, although mucosal surface area was increased as a result of villus hypertrophy, macromolecular leak appeared not to be increased. While this at first sight might indicate a tightened paracellular barrier, it may instead rather reflect the increased subepithelial diffusion barrier. Epithelial tight junctions represent the paracellular pore pathway. In the intestine, they are largely sodium selective [30], while the electrical resistance of the subepithelial loose tissue is nonselective. In order to specifically address changes in the epithelium, this permselectivity was measured using Na/Cl dilution potentials and was found to be unchanged in the adapted jejunum with hypertrophied villi. If the increase in the transmucosal electrical resistance was entirely due to the enlarged subepithelial intravillous tissue, one would expect a decreased sodium selectivity. But the permselectivity remained unchanged, indicating that the epithelial sodium permeability was increased in the adapted jejunum. This would facilitate paracellular back-leakage of absorbed sodium to enhance sodium–nutrient-coupled transport. Future studies will test if the transcellular transport route is enhanced in the adapted epithelium as well. This experimental SBS model in mice appears suitable for these studies because genetically manipulated mice facilitate testing of specific physiological processes.

In this study, we have analyzed a mouse model of 40% ICR that mimics moderate to severe human SBS. It is a suitable tool to study mechanisms of intestinal adaptation as well as to characterize genetic risk factors for intestinal failure. Both increased stool water content and villus elongation are hallmarks of this model. Our functional studies indicate that increased intestinal epithelial sodium permeability is a mechanism of epithelial adaptation that is active in addition to villus growth. Furthermore, hyperaldosteronism counteracts stool water loss in this murine extensive ileocecal resection model. Future studies are needed to address additional epithelial mechanisms such as transport function in the complex process of adaptation to severe SBS.

## Electronic supplementary material

Below is the link to the electronic supplementary material.

Supplementary material 1 (DOCX 16 kb)
