# Supplementary material for: Villus Growth, Increased Intestinal Epithelial Sodium Selectivity, and Hyperaldosteronism Are Mechanisms of Adaptation in a Murine Model of Short Bowel Syndrome
Source: Dig Dis Sci. 2018 Dec 20;64(5):1158–70. doi: 10.1007/s10620-018-5420-x (PMC6548203; doi:10.1007/s10620-018-5420-x)
Supplement: Supplementary file 1 — Supplementary material 1 (DOCX 16 kb) [file 10620_2018_5420_MOESM1_ESM.docx]

**Confidential Supplemental – Aldosterone Mass Spectrometry**

Steroids in mouse 500 µl serum samples were measured using MassChrom Steroids in Serum/Plasma Kit (Chromsystems, Germany). Calibrators were purchased from Chromsystems. Solid phase extraction sample preparation procedure was carried out like the manufacturers protocol. An Agilent 1290 UPLC series binary pump with 2x54 vial sample tray sampler with thermostat temperature controlled column compartment 2 position /10 ports switching valve was used.

*LC conditions*

| Source Parameter | Value | |  |
| --- | --- | --- | --- |
|  |  | |  |
| Column | Chromsystems order number: 72110 | |  |
| Mobile phase | A and B ( Chromsystems) | |  |
| Column temperature | 32°C | |  |
| Autosampler temperature | 4°C | |  |
| Injection volume | 10 µl | |  |
| Needle wash | 10 sec; Rinse Solution (Chromsystems) | |  |
|  |  | |  |
| Gradient |  | |  |
| Time in min | | Flow in ml/min | % Solvent B |
|  | |  |  |
| 2,00 | | 0,8 | 17 |
| 2.01 | | 0,8 | 33 |
| 3,40 | | 0,8 | 33 |
| 3,41 | | 0,4 | 33 |
| 4,70 | | 0,4 | 33 |
| 4,71 | | 0,4 | 37 |
| 6,50 | | 0,4 | 37 |
| 6,51 | | 0,6 | 37 |
| 9,50 | | 0,6 | 37 |
| 9,51 | | 0,6 | 100 |
| 10,50 | | 0,6 | 100 |
| 10,51 | | 0,8 | 17 |
| 11,50 | | 0,8 | 17 |
|  | |  |  |

The Agilent MS/MS system consisted of an Agilent 6490 Triple Quadruple Mass Spectrometer with Agilent MassHunter Software B 07.01. 10 µl was injected per run.

*MS conditions*

| Source Parameter | Value(-) |
| --- | --- |
|  |  |
| Ion mode | Agilent Jet Stream ESI+ |
| Gas temperature | 290°C |
| Gas flow (l/min) | 14 |
| Nebulizer (psi) | 25 |
| Sheath Gas Heater | 400 |
| Sheath Gas Flow | 11 |
| Capillary (V) | 4000 |
| VCharging | 1500 |
|  |  |

| Ion Funnel Parameters | Value |
| --- | --- |
|  |  |
| Pos High Pressure RF | 200 |
| Pos Low Pressure RF | 150 |
| Neg High Pressure RF | 110 |
| Neg Low Pressure RF | 60 |
|  |  |

| MRM transitions | Precursor (m/z) | Product (m/z) | Dwell | Frag (V) | CE (V) | Cell Acc (V) | Polarity |
| --- | --- | --- | --- | --- | --- | --- | --- |
|  |  |  |  |  |  |  |  |
| Aldosteron-d4 | 363 | 335,1 | 400 | 380 | 14 | 3 | negative |
| Aldosteron quant | 359 | 331,1 | 800 | 380 | 14 | 3 | negative |
| Aldosteron qual | 359 | 189 | 250 | 380 | 18 | 3 | negative |
|  |  |  |  |  |  |  |  |
|  |  |  |  |  |  |  |  |

*Data analysis*

Agilent MassHunter Quantitative Data Analysis Software (B.07.01) was used for data analysis. A 1/x^2^ weighting factor was applied during linear regression of the calibration curves. The quantitation using MassHunter Quantitative Software was performed by comparing chromatographic peak area ratio to a fixed concentration of the internal standard.
